# *Ficus pandurata* as a Functional Phytotherapeutic: Inhibiting JAK2/STAT3 Signaling and Activating Mitochondrial Apoptosis in Hepatocellular Carcinoma

**DOI:** 10.1002/fsn3.71114

**Published:** 2025-11-04

**Authors:** Muhammad Majid, Bing Tang, Yingyao Lai, Xiaoyan Pang, Yongdui Ruan, Hui Shi, Weiwen Peng, Weibo Dai, Xianjing Hu

**Affiliations:** ^1^ Dongguan Key Laboratory of Fundamental Research and Clinical Application of Toxic Chinese Medicine The First Dongguan Affiliated Hospital, Guangdong Medical University Dongguan P.R. China; ^2^ Guangdong Provincial Key Laboratory of Natural Drugs Research and Development Guangdong Medical University Dongguan P.R. China; ^3^ Dongguan Key Laboratory of TCM for Prevention and Treatment of Digestive Diseases Guangdong Medical University Dongguan P.R. China; ^4^ Pharmacology Laboratory Zhongshan Hospital of Traditional Chinese Medicine Affiliated to Guangzhou University of Traditional Chinese Medicine Zhongshan P.R. China; ^5^ Dongguan Branch National Engineering Research Center for Modernization of Traditional Chinese Medicine Dongguan P.R. China

**Keywords:** apoptosis, *Ficus pandurata*, functional food, hepatocellular carcinoma, JAK2/STAT3, nutraceutical

## Abstract

Chronic inflammation plays a key role in the development of hepatocellular carcinoma (HCC), one of the most prevalent and lethal forms of liver cancer. This study aimed to evaluate the anti‐HCC potential of the petroleum ether extract of *Ficus pandurata* Hance (FPHPE), a traditional hepatoprotective herb, focusing on its pro‐apoptotic and anti‐inflammatory actions via the JAK2/STAT3 signaling pathway. In vitro experiments demonstrated significant growth inhibition of HepG2, SMMC7721, and Hep3B cells following FPHPE treatment. GC–MS profiling identified 26 phytoconstituents, including friedelane, seseline, bergaptan, and tocopherols, many with known bioactivity. In a xenograft mouse model, FPHPE markedly suppressed tumor growth without causing systemic toxicity. Mechanistic analyses demonstrated that FPHPE activated mitochondria‐mediated apoptosis, as confirmed by Annexin V/PI flow cytometry, Western blot quantification from three biological replicates, TEM imaging showing disrupted cristae, and JC‐1 staining revealing mitochondrial membrane depolarization. Concurrently, FPHPE downregulated phosphorylated and total JAK2/STAT3, inhibited STAT3 nuclear translocation, and suppressed key downstream effectors (iNOS, COX2, c‐Myc, Vimentin, and Slug). ELISA further confirmed a reduction of pro‐inflammatory cytokines TNF‐α and IL‐1β in tumor tissues. Together, these findings establish FPHPE as a dual‐action phytotherapeutic candidate that interrupts both survival and inflammatory pathways, positioning *F. pandurata* as a promising source for nutraceuticals or complementary therapies against inflammation‐driven liver cancer.

## Graphical abstract

Triterpenoid‐ and coumarin‐rich *Ficus pandurata* Hance petroleum ether extract (FPHPE) significantly inhibited HepG2, SMMC7721, and Hep3B proliferation and suppressed tumor burden in xenograft mice without systemic toxicity. Comprehensive validation confirmed mitochondria‐mediated apoptosis through Annexin V/PI flow cytometry, Western blot, TEM visualization of cristae disruption, and JC‐1 detection of membrane depolarization. Mechanistically, FPHPE inhibited JAK2/STAT3 signaling, prevented STAT3 nuclear translocation, and downregulated oncogenic and inflammatory mediators (c‐Myc, iNOS, COX2, Vimentin, Slug, TNF‐α, IL‐1β), supporting its potential as a functional nutraceutical.

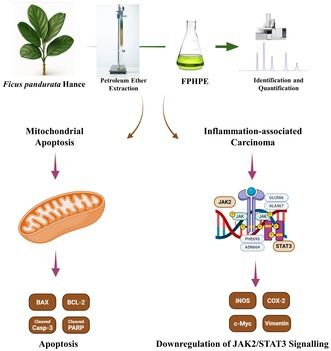

## 1 Introduction

The present‐day world is confronting hepatocellular carcinoma (HCC) as a pressing global health challenge, ranking among the leading causes of cancer‐related mortality and exhibiting a steadily rising incidence across both developed and developing regions (Choi et al. 2023; Sung et al. 2021). Despite significant strides in early detection and localized treatments such as liver resection and ablation, many patients are diagnosed at advanced stages when curative options are limited (Dai et al. 2024; Singal et al. 2023). Pharmacological therapies—including agents like sorafenib and lenvatinib—have extended survival for some, yet their impact is often tempered by drug resistance, off‐target effects, and systemic toxicity (Feng et al. 2020; Kudo et al. 2018). The reality remains sobering: global five‐year survival rates for HCC continue to fall below expectations (Feng et al. 2020). These shortcomings have fueled a growing interest in alternative strategies, particularly those rooted in natural products and plant‐based bioactives, which offer a multi‐targeted mode of action with potentially lower toxicity (Kazmi et al. 2018). Within this context, exploring dietary and traditional medicinal sources for liver‐protective and anticancer properties is both timely and necessary.

It is now well recognized that chronic inflammation is not merely a consequence of liver dysfunction but a driving force in hepatocarcinogenesis, linking underlying liver injury, immune dysregulation, and oncogenic signaling into a self‐perpetuating disease cycle. Inflammatory mediators—such as TNF‐α, IL‐1β, iNOS, and COX2—act as molecular accelerants of tumor initiation and progression by fostering genomic instability, promoting cellular proliferation, and suppressing tumor immune surveillance (Sgambato and Cittadini 2010). This chronic inflammatory milieu induces DNA damage, fosters genomic instability, upregulates proto‐oncogene expression, and suppresses tumor‐suppressor gene activity, thereby facilitating the malignant evolution of hepatocytes (Hibino et al. 2021; Karin and Shalapour 2022). Given its central role in tumor progression, inflammation represents a compelling therapeutic target in HCC. Several inflammatory signaling pathways—including IL‐17, iNOS, COX2, MAPKs, LPS/TLR4/MyD88/NF‐κB, and notably JAK2/STAT3—are critically implicated in this process (Gautam et al. 2021; Mandal et al. 2021). Among the key signaling cascades governing inflammation‐induced oncogenesis, the JAK2/STAT3 axis stands out for its central role in integrating cytokine signaling with pro‐tumorigenic transcriptional programs (Attia et al. 2021). JAK2, a cytoplasmic tyrosine kinase, phosphorylates STAT3 in response to upstream signals, enabling its dimerization and nuclear translocation (Hibino et al. 2021). Once inside the nucleus, STAT3 transcriptionally activates numerous oncogenic and inflammatory genes, including Bcl2, iNOS, COX2, c‐Myc, Vimentin, and Slug (Zhang et al. 2020). Aberrant and sustained activation of this pathway promotes cancer cell survival, immune evasion, and metastasis throughout the course of HCC development (Xiong et al. 2020; Zhou et al. 2021). Therefore, pharmacologically interrupting the JAK2/STAT3 signaling cascade emerges as a rational and promising strategy for developing anti‐HCC therapies that are capable of targeting both inflammation and tumor progression in an integrated manner.

Ethnopharmacology has long offered a reservoir of therapeutic innovation, particularly for inflammation‐associated diseases like HCC, where conventional treatments fall short. Among these natural remedies, traditional herbal medicines have demonstrated considerable potential due to their multitargeted actions, favorable safety profiles, and capacity to modulate inflammation and apoptosis. A growing body of evidence highlights that several medicinal plants not only suppress tumor growth but also enhance immune response and prolong survival in HCC patients (Cheng et al. 2016; Thu et al. 2018). *Ficus pandurata* Hance (FPH), a species of the Moraceae family and a widely used traditional Chinese medicinal herb, is esteemed in South China for its role in detoxification, blood circulation enhancement, and liver protection (Ramadan et al. 2009). Our previous studies have substantiated its pharmacological efficacy in mitigating acute alcohol‐induced hepatic injury and in alleviating colitis‐associated liver damage through anti‐inflammatory and antioxidative mechanisms (Dai, Chen, et al. 2021; Dai, Zhan, et al. 2021).

Despite its well‐established role in traditional medicine as a liver‐protective herb, the potential anticancer effects of FPH, particularly in HCC, remain largely unexplored within a scientific framework. This is a notable gap given the urgent demand for safer, more holistic interventions that can complement or enhance current therapies—especially those derived from dietary or plant‐based sources. To address this unmet need, the present study was designed with two major objectives: (i) to comprehensively evaluate the anti‐proliferative and tumor‐suppressive effects of different fractions of *F. pandurata* , with a specific focus on its petroleum ether fraction (FPHPE), in both in vitro and in vivo HCC models; and (ii) to elucidate the mechanistic basis of its activity, focusing on mitochondrial apoptosis induction and inhibition of the JAK2/STAT3 inflammatory axis. By combining phytochemical profiling with functional and mechanistic assessments, this work contributes to the growing body of evidence supporting plant‐derived agents as promising candidates in the development of functional or nutraceutical interventions for liver cancer.

## 2 Materials and Methods

### 2.1 Chemicals and Reagents

Dulbecco's Modified Eagle Medium (DMEM), fetal bovine serum (FBS) (Gibco, America, 42H9852K), penicillin, and streptomycin were obtained from Gibco Life Technologies (Grand Island, America, 8120364). Analytical grades of ethyl alcohol, petroleum, chloroform, ethyl acetate, and n‐butyl alcohol were obtained from Tianjin Damao Chemical Reagent Factory (Tianjin, China). Elisa kits including IL‐1β (370210818) and TNF‐α (569210825) were purchased from Tianjin Anoric Bio‐technology CO. Ltd. (Tianjin, China). The primary antibodies of cleaved PARP (AF7023), Bcl2 (AF6139), phospho‐JAK2 (AF3024), JAK2 (AF6022), and Bax (AF0120) were obtained from Affinity Biosciences Ltd. (OH, USA). Primary antibodies of cleaved caspase 3 (#9664), STAT3 (#9139), c‐Myc (#18583), α/β‐tubulin (#2148), histone (#4499), β‐actin (#12262), iNOS (#13120), and COX2 (#12282) were obtained from Cell Signaling Technology (Boston, USA). Primary antibodies of phospho‐STAT3 (sc‐8059) were obtained from Santa Cruz (California, USA).

### 2.2 Preparation for FPH Fractions

**FIGURE 1:**
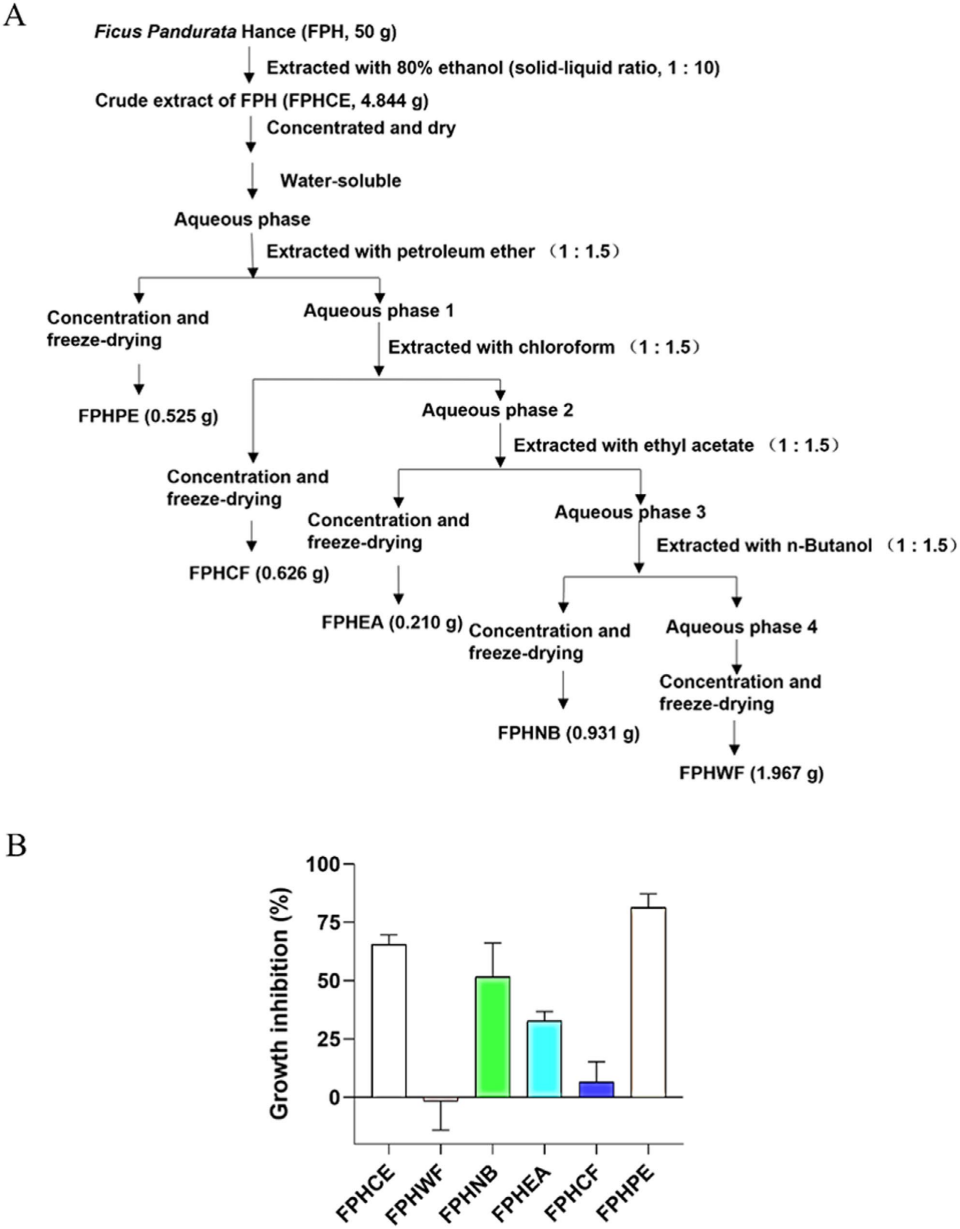
Extraction of different fractions of FPH and their effects on growth inhibition in HCC cells: (A) The extraction for different parts of FPH fraction, including petroleum ether extraction (FPHPE), chloroform extraction (FPHCF), ethyl acetate extraction (FPHEA), *n*‐butanol extraction (FPHNB), water extraction (FPHWF), and crude extract (FPHCE). (B) The growth inhibition of FPHCE, FPHWF, FPHNB, FPHEA, FPHCF, and FPHPE on HCC was determined by MTT assay. Results were expressed as mean ± SEM.

The whole plant was collected from Dajin Village, Kaiping City, Jiangmen City, Guangdong Province, as we reported previously (Dai, Chen, et al. 2021), and identified as *Ficus pandurata* Hance (FPH) by Professor Huang Haibo, Department of TCM Identification, Guangzhou University of Chinese Medicine. The dried FPH was pulverized and extracted with 80% ethanol (solid–liquid ratio 1:10) three times by heating under reflux. The crude extract of FPH was obtained by condensing and drying, and different fractions of FPH were obtained by extracting with petroleum ether, chloroform, ethyl acetate, and n‐butanol, successively. After concentrating and drying, the powders of petroleum ether extract (FPHPE), chloroform extract (FPHCF), ethyl acetate extract (FPHEA), n‐butanol extract (FPHNB), and water extract (FPHWF) of FPH were obtained, respectively (Figure 1A).

### 2.3 GC/MS Analysis

Gas chromatography–mass spectrometry (GC–MS) analysis was performed using an Agilent 7890A gas chromatograph paired with a 5975C mass selective detector system and an HP‐MS capillary column (60 m × 250 μm × 0.25 μm; Agilent Technologies). A 1 μL aliquot of the FPHPE sample was injected into the system at an injector temperature of 310°C. Nitrogen serves as the carriage gas, flowing at a rate of 1.5 mL/min. The oven temperature was programmed as follows: starting at 80°C and held for 2 min, then ramped to 180°C at 8°C/min and held for another 2 min. Subsequently, the temperature was increased to 250°C at 5°C/min and held for 2 min before a final rise to 310°C at 5°C/min, maintaining a temperature for 10 min. The analytes were detected in full‐scan mode, with a mass range of m/z 35–500 under electron impact (EI) conditions. The electron energy was set to 70 eV, and the ion source temperature was maintained at 230°C.

### 2.4 Cell Lines and Culture

The HepG2, SMMC7721, and Hep3B cell lines were sourced from the Shanghai Institute of Cell Biology, Chinese Academy of Sciences (Shanghai, China). These cells were cultivated in DMEM supplemented with 10% fetal bovine serum (FBS) and 1% antibiotic solution, containing 100 IU/mL penicillin and 100 μg/mL streptomycin. The cultures were maintained in a 37°C humidified incubator with 5% CO_2_.

### 2.5 Morphological Analysis

HepG2 and SMMC7721 cells were plated in 6‐well culture plates at a density of 4 × 10^4^ cells per well. After 24 h, the cells were treated with or without FPHPE for 48 h. Following treatment, the medium was removed, and the cells were washed with pre‐chilled PBS buffer. The morphological changes of the cells were then observed under an optical microscope (40×, Nikon Corporation, 108‐6290, Tokyo, Japan).

### 2.6 MTT Assay

Cells were seeded in 96‐well plates at a density of 3.5 × 10^3^ cells/well and treated with FPHPE at the concentrations of 7.81, 15.6, 31.2, 62.5, 125, 250 μg/mL for 48 h, as described previously (Pezzani et al. 2014). The absorbance of each well was detected, and the inhibition was calculated according to the following formula:

$$\text{Growth inhibition}=\left[1-\left(A_{\text{Sample}}-A_{\text{Blank}}\right)/\left(A_{\text{Control}}-A_{\text{Blank}}\right)\right]\times 100\%$$

The IC_50_ value was calculated accordingly. The experiment was performed in triplicate and repeated three times.

### 2.7 Western Blot

To extract total protein from cellular and tissue samples, a RIPA lysis buffer was employed in conjunction with mechanical homogenization. The protein concentration in the resulting extracts was measured using a BCA assay. Equal volumes of protein were then loaded onto SDS‐PAGE gels for electrophoresis, followed by transfer to PVDF membranes. After blocking the membranes to prevent non‐specific binding, they were incubated with primary antibodies specific to various target proteins, including cleaved caspase‐3, cleaved PARP, Bcl‐2, Bax, JAK2, STAT3, phosphorylated JAK2/STAT3, c‐Myc, β‐actin (serving as a loading control), COX‐2, iNOS, Vimentin, and Slug. After this, HRP‐conjugated secondary antibodies were applied, and the protein bands were detected using ECL. The intensity of the bands was quantified with ImageJ software for further analysis.

### 2.8 Extraction for Nuclear and Cytoplasmic Proteins

Cellular fractionation was performed to isolate nuclear and cytoplasmic protein fractions. A commercial kit (Nuclear and Cytoplasmic Protein Extraction Kit, P0027, Beyotime Biotechnology, Shanghai, China) was utilized according to the manufacturer's instructions. Briefly, cells were harvested, washed with PBS, and pelleted by centrifugation. Cytoplasmic proteins were extracted using a two‐step lysis buffer system, followed by centrifugation to remove cellular debris. Subsequently, nuclear proteins were extracted from the remaining pellet using a dedicated nuclear extraction buffer. Protein concentrations were determined, and samples were denatured and subjected to Western blot analysis to assess the expression levels of STAT3, α/β‐tubulin (a cytoplasmic marker), and histone H3 (a nuclear marker).

### 2.9 Flow Cytometry Analysis

HepG2 and SMMC7721 cells were cultured in 6‐well plates, with a seeding density of 2.5 × 10^5^ cells per well. After 24 h of incubation, various concentrations of FPHPE (15.6, 31.2, 62.5, 125, and 250 μg/mL) were applied to the cells for 48 h. Upon completion of the treatment, the cells were harvested, followed by centrifugation at 500 *g* for 5 min. The supernatant was discarded, and the cell pellet was washed twice with PBS to remove any residual media. Apoptotic cell rates were evaluated using flow cytometry, utilizing the Annexin V‐FITC/PI Apoptosis Assay kit (21103589, Biosharp, Hefei, China), following the manufacturer's instructions (Wang et al. 2020).

### 2.10 Transmission Electron Microscopy (TEM) Analysis

SMMC7721 cells were treated with FPHPE (0, 62.5, and 125 μg/mL) for 48 h, harvested, and fixed with 2.5% glutaraldehyde in 0.1 M phosphate buffer at 4°C overnight. Samples were post‐fixed with 1% osmium tetroxide, dehydrated through a graded ethanol series, and embedded in epoxy resin. Ultrathin sections (70 nm) were cut, stained with uranyl acetate and lead citrate, and examined using a transmission electron microscope (Hitachi H‐7650, Tokyo, Japan). Ultrastructural changes in mitochondria, including cristae morphology and membrane integrity, were analyzed (Yu et al. 2024).

### 2.11 JC‐1 Mitochondrial Membrane Potential Assay

Mitochondrial membrane potential (ΔΨm) was assessed using the JC‐1 assay kit (Beyotime, Shanghai, China) according to the manufacturer's instructions. Briefly, SMMC7721 cells were treated with FPHPE (0, 31.2, 62.5, and 125 μg/mL) for 48 h, incubated with JC‐1 working solution (5 μg/mL) at 37°C for 20 min, and washed twice with PBS. Fluorescence was detected using a fluorescence microscope (Olympus IX71, Tokyo, Japan). Red fluorescence (JC‐1 aggregates) indicated intact ΔΨm, while green fluorescence (JC‐1 monomers) indicated depolarization. The aggregate/monomer fluorescence ratio was quantified using ImageJ software (Shah and Dobrovolskaia 2024).

### 2.12 Establishment of HCC‐Bearing Xenograft Mouse Model and Drug Treatment

Nude (BALB/c nu/nu) male mice (20 ± 2 g) were supplied by Zhejiang Weitong Lihua Laboratory Animal Technology Co., LTD (Jiaxing, China). All mice were housed under a 12‐h light/dark cycle with ad libitum access to food and water. This Animal Experiment Protocol has been reviewed by the Laboratory Animal Ethics Committee of Zhongshan Hospital of Traditional Chinese Medicine. It is in line with the principles of animal protection, animal welfare, and ethics, and in line with the relevant provisions of the national laboratory animal ethics and welfare (Approval number: AEWC‐2021057). The mice received a subcutaneous inoculation of SMMC7721 cells with 8 × 10^5^ cells/mouse. Once the average tumor volume reached ~50 mm^3^, mice were randomly assigned to four groups based on tumor size and body weight, including the normal saline group (CMC‐Na), sorafenib group (ig, 20 mg/kg), FPHPE low dosage (FPHPE‐L, suspended in CMC‐Na, ig, 125 mg/kg) group, and FPHPE high dosage (FPHPE‐H, suspended in CMC‐Na, ig, 250 mg/kg) group. The body weights of mice were recorded every day, and tumor volumes were monitored every three days. After the treatment was completed, the mice were anesthetized with isoflurane and then euthanized for dissection. Blood was harvested, and serum was isolated by centrifugation at 4000 rpm for 10 min at 4°C. The organs included kidney, liver, lung, heart, and spleen, and tumor tissue were removed and kept at −80°C for subsequent analysis.

### 2.13 Statistical Analysis

Data are presented as the mean ± standard error of the mean (SEM). For the statistical assessment, one‐way ANOVA was utilized to analyze differences among multiple groups, followed by post hoc tests for pairwise comparisons. A *p*‐value of less than 0.05 was considered statistically significant.

## 3 Results

### 3.1 Anti‐HCC Screening of FPH Fractions

To dissect the therapeutic landscape of *Ficus pandurata* Hance and pinpoint its most potent anti‐HCC component, we systematically fractionated the crude ethanol extract into petroleum ether (FPHPE), chloroform (FPHCF), ethyl acetate (FPHEA), n‐butanol (FPHNB), and water (FPHWF) fractions. Each was subjected to an MTT‐based viability assay in HepG2 cells—a widely accepted model for hepatocellular carcinoma. The results were striking. Among all tested fractions, FPHPE exhibited the most pronounced cytotoxic effect, markedly suppressing HepG2 cell viability compared to its counterparts (Figure 1B). This prominent activity suggests that the bioactive phytoconstituents driving the anticancer potential of FPH are highly concentrated within its lipid‐soluble petroleum ether phase. This pivotal finding serves not only as the gateway to our subsequent investigations but also reinforces the rationale behind selecting FPHPE for comprehensive mechanistic dissection. Its dominant effect positions FPHPE as a leading candidate for the development of novel phytotherapeutic strategies against liver cancer, aligning squarely with our initial study objectives.

### 3.2 Fingerprinting of Phytoconstituents in FPHPE Using GC‐MS

**FIGURE 2:**
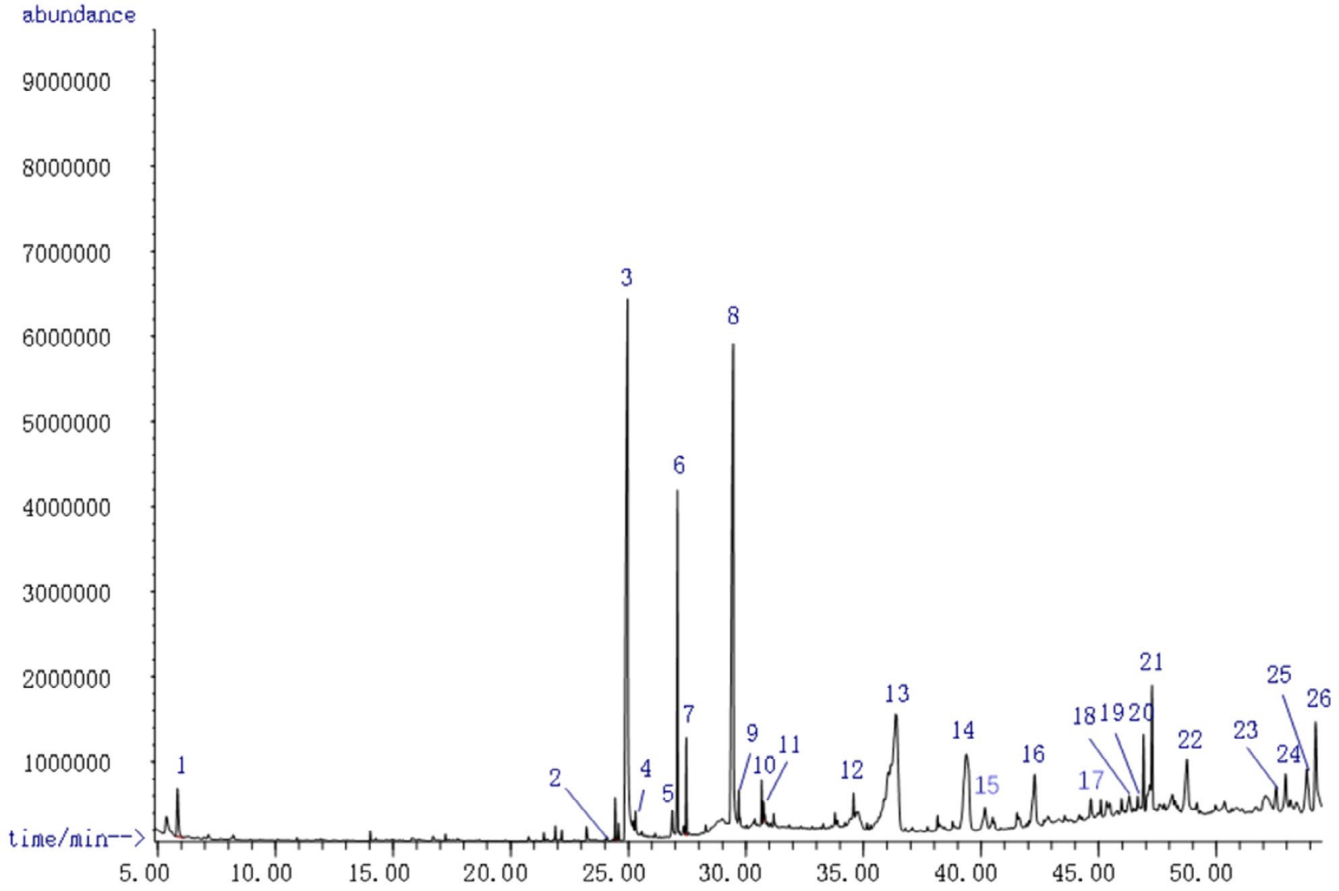
Total ion chromatograph of FPHPE. GC/MS was used to identify the main components of FPHPE, and 26 compounds in all were identified.

GC–MS analysis was performed to identify the key components of FPHPE. A total of 26 compounds were successfully identified through comparison with self‐conducted volatile component data and NIST17 (Figure 2). The detectable components of FPHPE, as analyzed by AMDIS, are represented in Table 1. Some of the major detected compounds include 4‐methyl‐3‐penten‐2‐one, phytol, ficusin, DIBP, 3,7,11,15‐tetramethyl‐2‐hexadecen‐1‐ol, hexadecanoic acid, butyl phthalate, ethyl palmitate, bergaptan, 8,8‐dimethyl‐2H,8H‐benzo (1,2‐b:3,4‐b’) dipyran‐2‐one, ethyl linoleate, ethyl oleate, 4,8,12,16‐tetramethylheptadecan‐4‐olide, (+)‐3‐oxo‐urs‐12‐en‐24‐oic acid methyl ester, friedelan‐3‐one, triacontane, moretenol acetate, delta‐tocopherol, and gamma‐tocopherol.

**TABLE 1:** Identified chemical constituents and their % content from FPHPE by GC/MS.

No.	Retention time (min)	Name	CAS no.	Relative content (%)
1	5.3653	3‐Penten‐2‐one, 4‐methyl	000141‐79‐7	0.8039
2	21.901	Myristaldehyde	000124‐25‐4	0.2655
3	24.4459	Phytol	000150‐86‐7	0.5344
4	24.5946	2‐Pentadecanone, 6,10,14‐trimethyl—	000502‐69‐2	0.2429
5	24.9691	Ficusin	000066‐97‐7	19.3157
6	25.2129	DIBP (Diisobutyl phthalate)	000084‐69‐5	0.3314
7	25.3081	3,7,11,15‐Tetramethyl‐2‐hexadecen‐1‐ol	102608‐53‐7	0.4503
8	26.8719	Hexadecanoic acid	000057‐10‐3	0.6491
9	27.0978	Butyl phthalate	000084‐74‐2	4.9974
10	27.4724	Ethyl palmitate	000628‐97‐7	1.2638
11	28.287	2H,8H‐Benzo[1,2‐b:5,4‐b]dipyran‐2‐one, 8,8‐dimethyl‐(Xanthyletin)	000523‐59‐1	0.1291
12	28.8459	Olean‐12‐en‐3‐ol, acetate, (3β)	001616‐93‐9	0.7845
13	29.4524	Bergaptan (5‐Methoxypsoralen)	000484‐20‐8	15.0955
14	29.6962	2H,8H‐Benzo[1,2‐b:3,4‐b] pyran‐2‐one, 8,8‐dimethyl‐(Seseline)	000523‐59‐1	0.647
15	30.6713	Ethyl linoleate	000544‐35‐4	0.6094
16	30.7546	Ethyl oleate	000111‐62‐6	0.3311
17	30.8081	Ethyl linolenate	001191‐41‐9	0.206
18	31.1827	Ethyl stearate	000111‐61‐5	0.2645
19	34.5778	4,8,12,16‐Tetramethylheptadecan‐4‐olide	096168‐15‐9	0.4058
20	36.3795	Urs‐12‐en‐24‐oic acid, 3‐oxo‐, methyl ester, (+)—	020475‐86‐9	17.2351
21	38.1454	Bis(2‐ethylhexyl) phthalate	000117‐81‐7	0.2909
22	39.3644	Friedelan‐3‐one	000559‐74‐0	6.6109
23	40.4881	Triacontane	000638‐68‐6	0.4852
24	42.2719	Moretenol acetate	002085‐25‐8	2.8887
25	44.6563	Delta‐Tocopherol	000119‐13‐1	0.8158
26	46.2854	Gamma‐Tocopherol	007616‐22‐0	0.6706

### 3.3 FPHPE Exhibits Potent Anti‐Proliferative Activity in HCC Cells In Vitro

**FIGURE 3:**
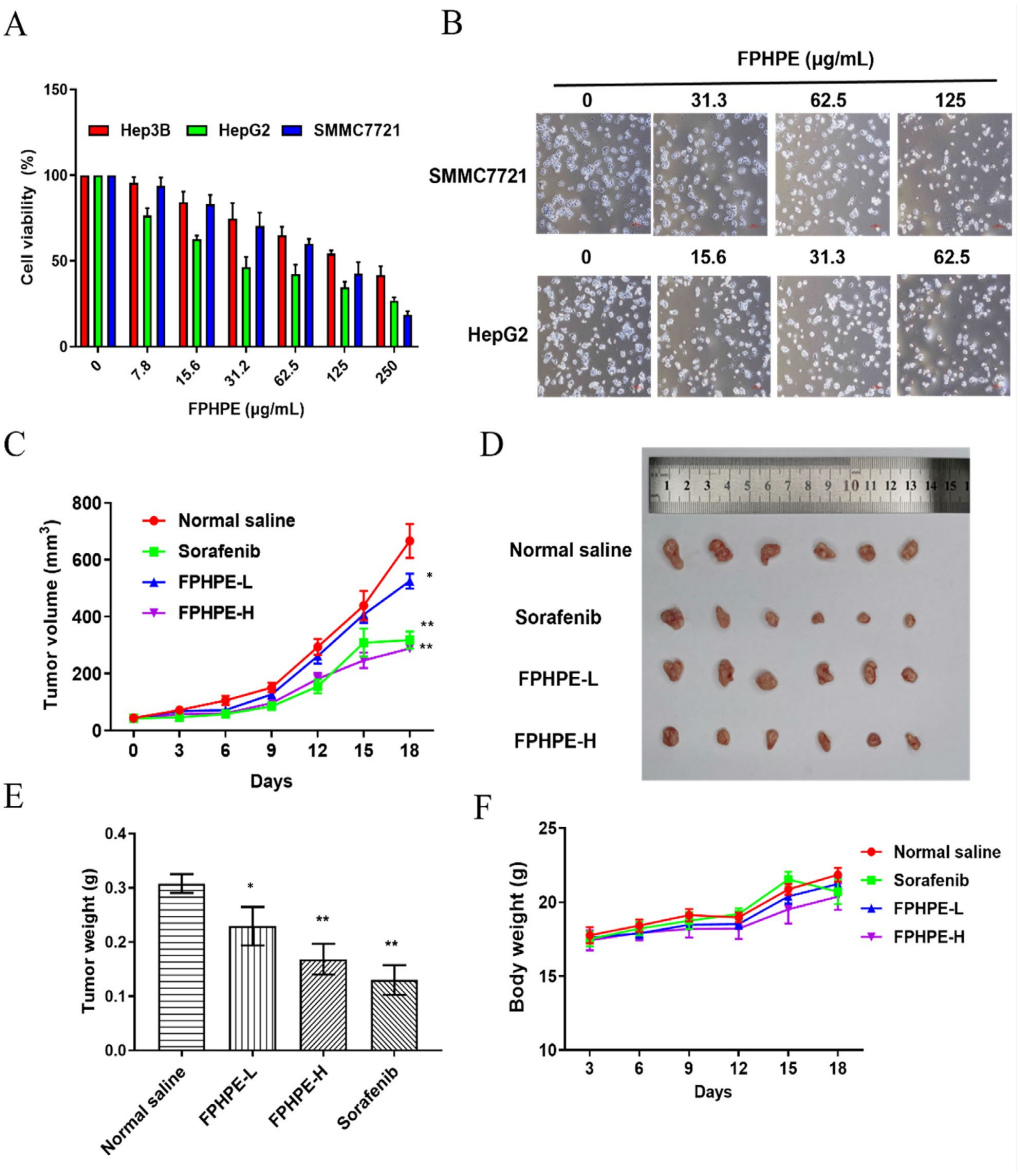
FPHPE inhibited HCC growth in vitro and in vivo. (A) FPHPE inhibited HCC cell growth in vitro. HCC cell lines, including Hep3B, HepG2, and SMMC7721, were treated with FPHPE at different concentrations for 48 h, and the cell viability was detected by MTT assay. (B) Effect of FPHPE on cell morphology. HepG2 and SMMC7721 cells were treated with FPHPE at different concentrations for 48 h, and the cells' morphological changes were evaluated by an optical microscope (40×). (C–F) FPHPE suppressed tumor growth in nude mice bearing SMMC7721 cells xenograft model. The effect of FPHPE on tumor volume (C), tumor size (D), tumor weight (E), and body weight (F). Results were expressed as mean ± SEM. *p < 0.05, **p < 0.01, versus normal saline group.

To validate the cytotoxic potential of FPHPE, we assessed its inhibitory effects on three HCC cell lines—HepG2, SMMC7721, and Hep3B—using the MTT assay. FPHPE exhibited robust, dose‐dependent growth inhibition in all tested lines, with IC50 values of 36.53 μg/mL (HepG2), 76.89 μg/mL (SMMC7721), and 161.37 μg/mL (Hep3B), respectively (Figure 3A). Morphological examination of treated cells revealed hallmark features of apoptosis, including cell shrinkage, membrane blebbing, and chromatin condensation (Figure 3B). These findings suggest that FPHPE exerts a pronounced cytotoxic effect on liver cancer cells, justifying its further evaluation in animal models.

### 3.4 FPHPE Suppresses Tumor Progression in HCC Xenograft Model

To validate the in vitro findings, we next examined the anti‐tumor efficacy of FPHPE in vivo using an immunodeficient mouse model bearing SMMC7721‐derived xenografts. As shown in Figure 3C,D, tumor volume and size were significantly reduced in both low‐dose (125 mg/kg) and high‐dose (250 mg/kg) FPHPE‐treated groups compared to the control. FPHPE reduced tumor weight in a dose‐dependent manner, with values of 0.23 ± 0.04 g (low dose) and 0.17 ± 0.03 g (high dose), compared to 0.31 ± 0.02 g in the control group (*p* < 0.01) (Figure 3E). Importantly, no significant body weight loss was observed throughout the treatment period (Figure 3F), underscoring the safety and tolerability of FPHPE. Together, these in vitro and in vivo findings establish FPHPE as a promising multi‐potent agent capable of suppressing HCC growth with high efficacy and minimal toxicity, warranting further investigation as a therapeutic candidate.

### 3.5 FPHPE Suppressed HCC Growth by Inducing Mitochondria‐Mediated Apoptosis

**FIGURE 4:**
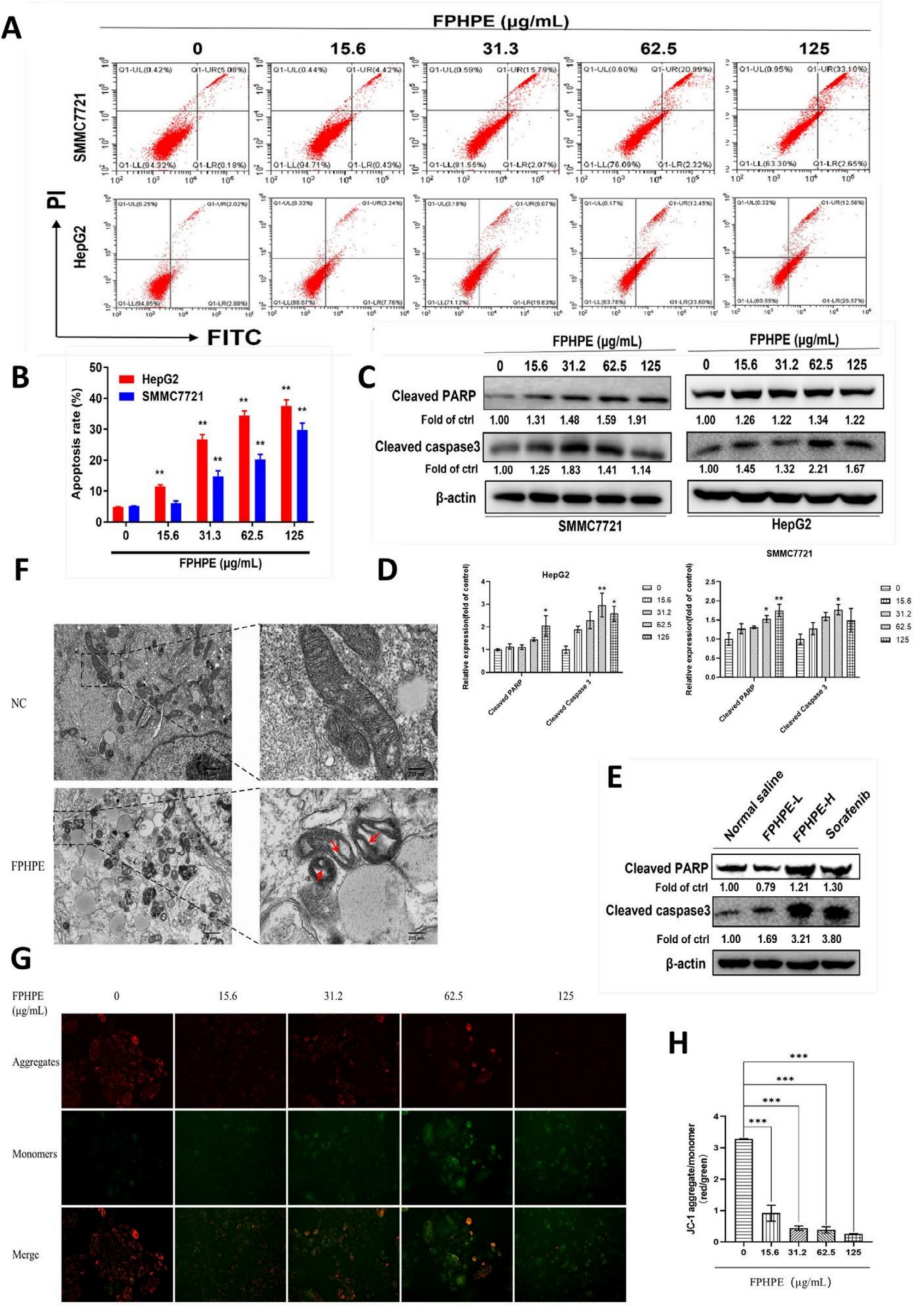
FPHPE induced mitochondria‐mediated apoptosis in HCC cells. (A, B) FPHPE increased apoptosis rates in HepG2 and SMMC7721 cells. Cells were treated with FPHPE at the indicated concentrations for 48 h, and apoptotic rates were assessed by Annexin V‐FITC/PI flow cytometry (A) with quantitative analysis shown in (B). (C, D) FPHPE enhanced the expression of apoptosis‐associated proteins. Western blot analysis of cleaved PARP and cleaved caspase‐3 in HCC cell lines (C) and tumor tissues (E), with densitometric quantification of three independent replicates shown in (D). β‐Actin served as the loading control. Results are expressed as mean ± SEM (*p < 0.05, **p < 0.01 vs. control). (F) Transmission electron microscopy (TEM) revealed ultrastructural changes in mitochondria. Control (NC) cells displayed intact mitochondria with well‐organized cristae, whereas FPHPE‐treated cells exhibited disrupted cristae, mitochondrial swelling, and vacuolization (red arrows), characteristic of mitochondrial apoptosis. (G, H) JC‐1 staining demonstrated mitochondrial membrane potential (ΔΨm) disruption. Control cells showed predominant red fluorescence (JC‐1 aggregates, intact ΔΨm), while FPHPE treatment induced a concentration‐dependent shift to green fluorescence (JC‐1 monomers), indicating mitochondrial depolarization. Representative images are shown in (G), and quantitative analysis of aggregate‐to‐monomer ratios is presented in (H). Data are shown as mean ± SEM (***p < 0.001 vs. control).

Apoptosis is a fundamental mechanism underlying the efficacy of anticancer agents (Pistritto et al. 2016). To investigate whether the cytotoxic effects of FPHPE were mediated by apoptosis, HepG2 and SMMC7721 cells were subjected to Annexin V‐FITC/PI staining. Flow cytometric analysis revealed a significant, dose‐dependent increase in both early and late apoptotic populations following FPHPE treatment (Figure 4A,B), indicating that apoptosis plays a central role in its anti‐HCC activity. To further validate this observation, we examined the expression of apoptosis‐related proteins by Western blot. FPHPE treatment markedly enhanced the cleavage of caspase‐3 and its downstream substrate PARP, while also elevating the Bax/Bcl‐2 ratio in both HCC cell lines and tumor tissues (Figure 4C–E). These molecular events are hallmarks of activation of the intrinsic mitochondrial apoptotic pathway. Ultrastructural analysis using transmission electron microscopy (TEM) provided additional confirmation at the organelle level. Control cells exhibited intact mitochondrial membranes with dense cristae, whereas FPHPE‐treated cells displayed swollen mitochondria, disrupted cristae, and vacuolization (Figure 4F), consistent with mitochondrial damage during apoptosis. Finally, we assessed mitochondrial membrane potential (ΔΨm) using JC‐1 staining. Control cells exhibited predominantly red fluorescence (JC‐1 aggregates, intact ΔΨm), whereas FPHPE treatment induced a concentration‐dependent shift from red to green fluorescence (JC‐1 monomers), reflecting marked mitochondrial depolarization (Figure 4G,H). Taken together, these complementary findings—ranging from flow cytometry and protein expression to ultrastructural and functional mitochondrial assessments—provide compelling evidence that FPHPE induces apoptosis in HCC cells primarily through the intrinsic mitochondrial pathway, characterized by mitochondrial depolarization, cristae disruption, caspase activation, and modulation of Bcl‐2 family proteins. These results strongly support the mechanistic relevance of FPHPE's pro‐apoptotic activity and reinforce its potential as a phytotherapeutic agent against liver cancer.

### 3.6 FPHPE Inhibited HCC Growth via Suppressing JAK2/STAT3 Pathway

**FIGURE 5:**
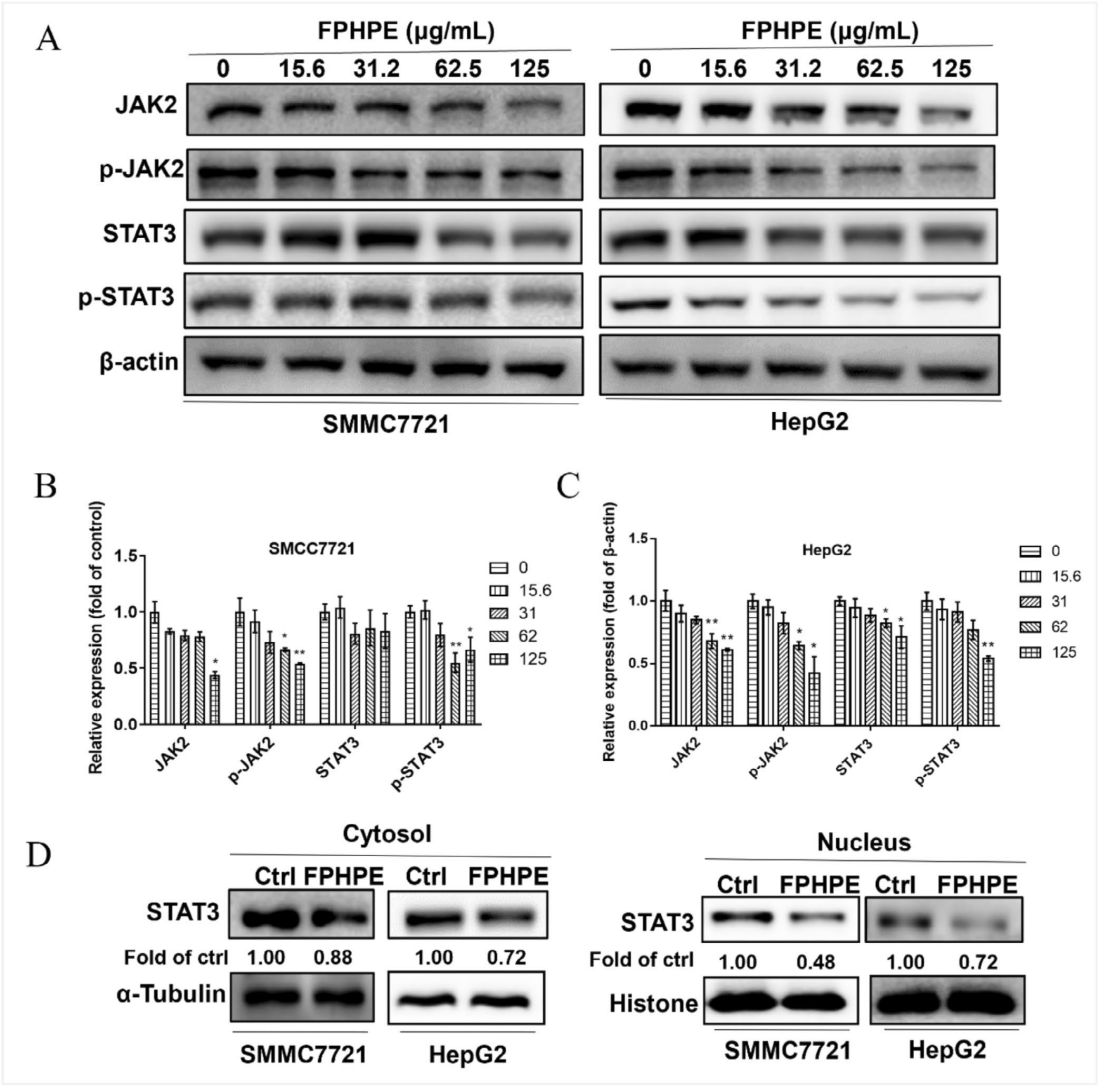
FPHPE inhibited HCC growth by suppressing the JAK2/STAT3 pathway. (A–C) Effect of FPHPE on the protein expressions of the JAK2/STAT3 pathway. HepG2 and SMMC7721 cells were treated with FPHPE at the indicated concentrations for 48 h, and the expressions of JAK2, phospho‐JAK2, STAT3, and phospho‐STAT3 were detected via western blot assay (A) and quantitative analysis by normalizing to β‐Actin (B, C). (D) FPHPE suppressed the JAK2/STAT3 pathway by inhibiting the translocation of STAT3 from the cytoplasm to the nucleus. HepG2 and SMMC7721 were treated with FPHPE for 48 h, and the expression of STAT3 in the cytoplasm and nucleus was detected via western blot assay. Results were expressed as mean ± SEM. *p < 0.05, **p < 0.01, versus the control group.

Cancer‐associated inflammation plays a remarkable role in the progression of HCC, encompassing its initiation, growth, invasion, and metastasis. These inflammatory responses lead to the overproduction of numerous cytokines and active mediators' whim, which in turn amplify the formation and remodeling of the tumor microenvironment (Nagai et al. 2021). JAK2/STAT3 is a classical regulatory pathway for the proliferation and apoptosis of malignant tumor cells. It is reported that the abnormally activated JAK2/STAT3 signaling pathway enhances the occurrence and development of HCC by promoting tumor cell proliferation, migration, invasion, angiogenesis, and apoptosis inhibition (Kang et al. 2021). In light of the pivotal role of inflammation in driving hepatocellular carcinoma progression, we next sought to determine whether the anti‐cancer effect of FPHPE involves modulation of the JAK2/STAT3 pathway—a central mediator of tumor‐associated inflammation and survival. Western blot analysis revealed that FPHPE significantly downregulated both total and phosphorylated forms of JAK2 and STAT3 in HepG2 and SMMC7721 cells in a dose‐dependent manner (Figure 5A–C). This observation points to effective suppression of pathway activation at both the kinase and transcription factor levels. To investigate the impact on STAT3‐dependent transcriptional activity, we performed subcellular fractionation to evaluate STAT3 nuclear localization. The results showed a clear reduction in nuclear STAT3 content following FPHPE treatment, indicating impaired translocation and transcriptional inactivation of key oncogenic and inflammatory genes (Figure 5D). These findings provide strong mechanistic evidence that FPHPE disrupts the JAK2/STAT3 signaling cascade, a known driver of cell survival, immune evasion, and metastasis in HCC. By targeting this pathway, FPHPE not only curtails pro‐tumorigenic signaling but also enhances the therapeutic relevance of inflammation suppression as a strategy in liver cancer management. Together, these data substantiate the hypothesis that FPHPE exerts anti‐inflammatory and anti‐tumor effects through targeted inhibition of the JAK2/STAT3 signaling cascade, reinforcing its potential as a dual‐action therapeutic agent in HCC management.

### 3.7 FPHPE Suppressed HCC Growth by Regulating STAT3 Downstream Targets and Inflammatory Mediators

**FIGURE 6:**
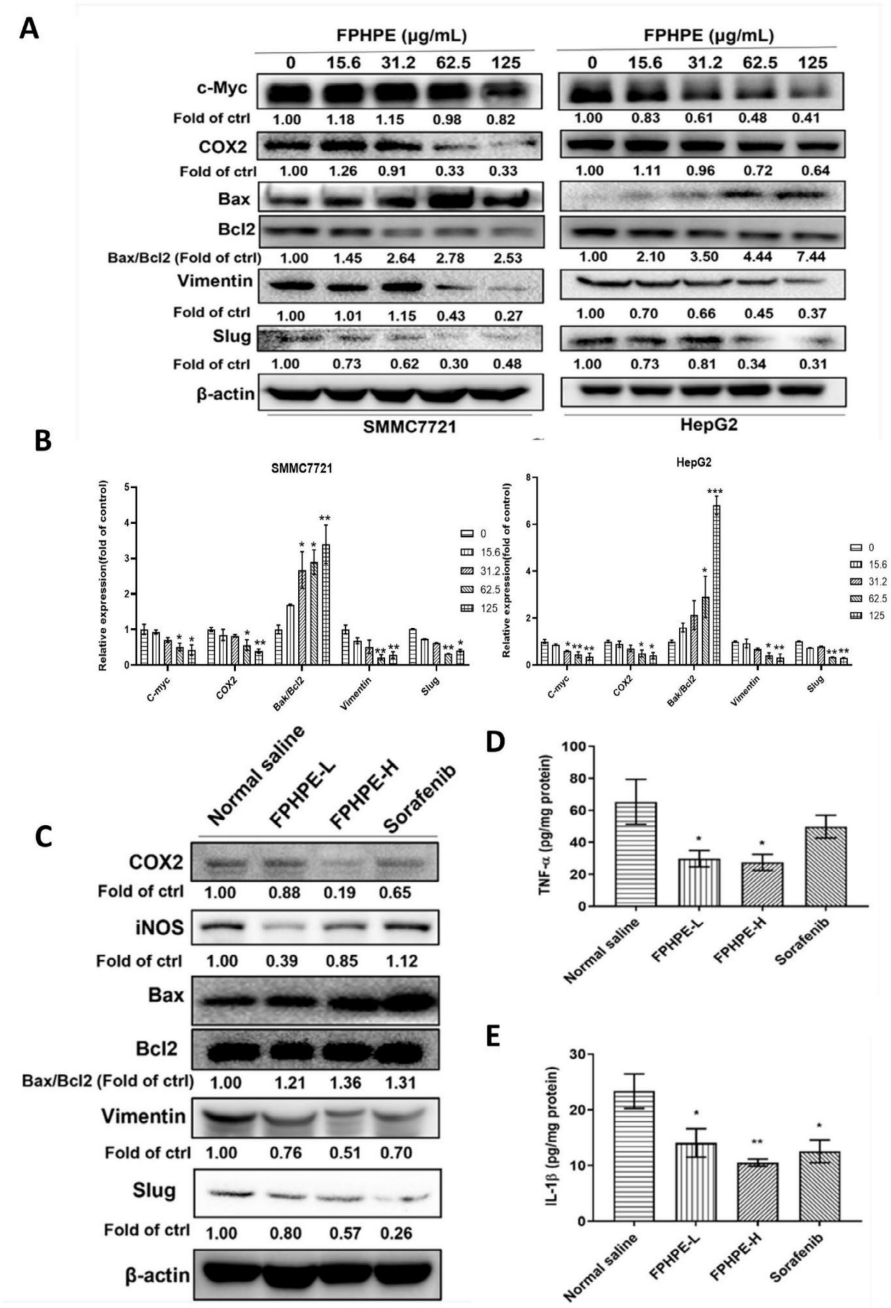
FPHPE suppressed HCC progression by regulating STAT3 downstream targets and inflammatory mediators. (A) Western blot analysis of c‐Myc, COX2, Bax, Bcl2, Vimentin, and Slug expression in HepG2 and SMMC7721 cells treated with increasing concentrations of FPHPE (0–125 μg/mL) for 48 h. Representative blots and fold‐change values relative to control (normalized to β‐actin) are shown. (B) Quantification of protein expression changes in SMMC7721 and HepG2 cells from three independent experiments. Data are expressed as mean ± SEM (*p < 0.05, **p < 0.01, ***p < 0.001 vs. control). (C) Western blot analysis of iNOS, COX2, Bax, Bcl2, Vimentin, and Slug expression in tumor tissues from xenograft‐bearing mice treated with FPHPE (low dose, high dose) or sorafenib for 18 days. Fold changes relative to control are indicated. (D, E) ELISA quantification of TNF‐α (D) and IL‐1β (E) levels in tumor tissues after treatment. Results are expressed as mean ± SEM (*p < 0.05, **p < 0.01 vs. normal saline group).

To further elucidate the molecular consequences of JAK2/STAT3 inhibition, we assessed the expression of downstream effectors involved in tumor proliferation, inflammation, apoptosis, and metastasis. Western blot analysis demonstrated that FPHPE treatment markedly reduced the expression of STAT3‐regulated oncogenic and inflammatory proteins, including c‐Myc, COX2, iNOS, Vimentin, and Slug, while concomitantly increasing the pro‐apoptotic marker Bax and elevating the Bax/Bcl2 ratio in both HepG2 and SMMC7721 cells (Figure 6A,B). Consistent with these in vitro findings, analysis of xenograft tumor tissues revealed a similar downregulation of COX2, iNOS, Vimentin, and Slug, together with an upregulation of Bax and suppression of Bcl2, further supporting the induction of mitochondria‐mediated apoptosis by FPHPE (Figure 6C). Moreover, ELISA quantification confirmed a significant decrease in the pro‐inflammatory cytokines TNF‐α and IL‐1β in tumor tissues following FPHPE administration (Figure 6D,E). Collectively, these results demonstrate that FPHPE exerts a dual inhibitory effect on hepatocellular carcinoma progression by (i) silencing STAT3 transcriptional output to suppress oncogenic, inflammatory, and metastatic pathways, and (ii) promoting mitochondrial apoptosis through Bax/Bcl2 modulation. This integrated mechanism reinforces the therapeutic potential of FPHPE as a credible phytotherapeutic candidate for liver cancer management.

## 4 Discussion

**FIGURE 7:**
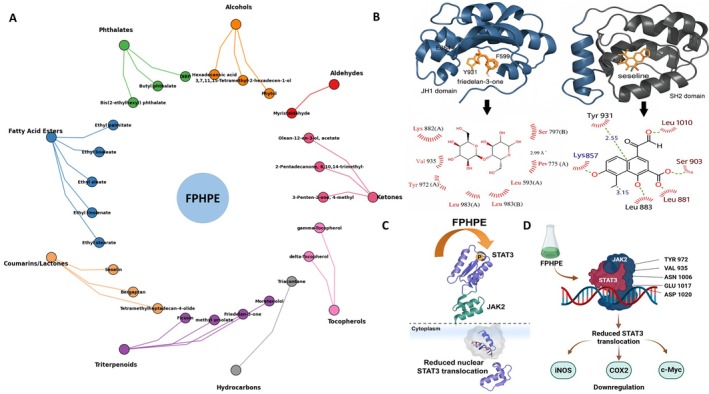
Mechanistic illustration of FPHPE‐induced anti‐HCC effects via phytochemical mapping, molecular docking, and pathway inhibition targeting JAK2/STAT3 signaling. (A) GC–MS‐based phytochemical fingerprinting of the petroleum ether fraction of FPHPE, showing class‐wise distribution of active compounds including coumarins/lactones, fatty acid esters, alcohols, phthalates, ketones, triterpenoids, tocopherols, and others. (B) Docking simulations demonstrate strong binding affinities of GCMS‐identified FPHPE compounds (friedelan‐3‐one and seselin) within the ATP‐binding site of the JH1 domain of JAK2 and the SH2 domain of STAT3. Top left: Friedelan‐3‐one shown embedded within the ATP‐binding pocket of the JAK2 JH1 domain (with interacting residues L595, F599, Y931, V863, E864). Top right: Seselin docked into the SH2 domain of STAT3, forming stable interactions. Bottom panels: 2D ligand interaction diagrams detailing hydrogen bonding and hydrophobic contacts with key residues (e.g., Tyr931, Lys857, Leu1010, Ser903, Leu883). (C) Schematic representation of FPHPE‐mediated inhibition of STAT3 phosphorylation and translocation. The JAK2/STAT3 complex is shown ti be disrupted in the cytoplasm, preventing nuclear entry of p‐STAT3. (D) Molecular consequence of FPHPE activity: Inhibition of JAK2 by FPHPE phytochemicals prevents STAT3‐mediated transcriptional activation. Key residues (TYR972, VAL935, ASN1006, GLU1017, ASP1020, etc.) are identified as critical contact points within JAK2. Resultant transcriptional silencing of downstream targets iNOS, COX2, and c‐Myc underpins the extract's anti‐inflammatory and anti‐oncogenic action in HCC.

Liver cancer remains a formidable global health challenge, with hepatocellular carcinoma (HCC) representing its most common and aggressive form. Despite the availability of surgical resection, ablation techniques, and targeted agents like sorafenib and lenvatinib, the prognosis for advanced HCC remains dismal—particularly in regions such as China, which accounts for nearly 47% of global liver cancer cases (Freddie Bray et al. 2018; Sung et al. 2021). Most patients are diagnosed at a late stage, and median survival often falls below six months (Bray et al. 2018). While immunotherapies, including PD‐1 inhibitors, have shown promise, their clinical benefit is limited to a small subset of patients, and resistance frequently develops (Rutherford et al. 2021). This therapeutic stagnation underscores an urgent need to explore novel, safe, and multi‐targeted treatment strategies. Unlocking new therapeutic strategies for HCC demands more than incremental advances—it requires bold exploration of novel molecular targets and untapped natural resources. Ethnopharmacology offers a rich, often underexplored, repository of bioactive compounds with multitargeted therapeutic potential. Among these, traditional Chinese herbal medicines have long held dual medicinal and nutritional value, and are commonly used not only in daily health maintenance but also in treating diseases marked by chronic inflammation and oxidative stress (Zhou et al. 2018). *F. pandurata* , a plant extensively used in South China for detoxification, blood circulation, and liver protection, has been highlighted in historical medical texts such as the *Shennong Classic of Materia Medica*, where numerous botanicals were credited with anti‐tumor properties (Zhou 2015). Building upon this historical and ethnomedical foundation, we investigated the petroleum ether fraction of FPH (FPHPE) for its potential anti‐HCC properties. Our results present a compelling translational shift from traditional remedy to mechanistically defined phytotherapy—substantiated by rigorous in vitro and in vivo evidence. This journey from folk medicine to pharmacological relevance reinforces the value of re‐examining ancient knowledge through the lens of modern biomedical science. A particularly noteworthy strength of this study lies in the multi‐layered confirmation of mitochondrial apoptosis induced by FPHPE. While many phytotherapy studies rely solely on flow cytometry or Western blotting, our work integrates four complementary lines of evidence: (i) Annexin V/PI flow cytometry demonstrating dose‐dependent apoptotic induction, (ii) Western blotting showing cleavage of caspase‐3 and PARP with an elevated Bax/Bcl‐2 ratio, (iii) TEM ultrastructural imaging revealing cristae disruption and mitochondrial swelling, and (iv) JC‐1 staining directly confirming mitochondrial depolarization. Taken together, these findings present one of the most comprehensive demonstrations of mitochondria‐mediated apoptosis reported for a natural product extract in HCC models. This integrated approach not only validates the mechanistic pathway with high confidence but also elevates the translational relevance of FPHPE as a phytotherapeutic candidate for liver cancer, setting a new benchmark for apoptosis‐focused investigations of plant‐derived agents. Chronic inflammation is now well‐established as a key enabler of tumorigenesis across multiple organ systems, with clear clinical correlations such as gastritis to gastric cancer, enteritis to colorectal cancer, and hepatitis to hepatocellular carcinoma (Batool et al. 2017; Khalyfa et al. 2021; Ndegwa et al. 2020; Zhu et al. 1997). Inflammation fuels the cancer continuum by promoting genetic instability, upregulating oncogene expression, suppressing tumor suppressors, and recruiting pro‐tumor immune cells that support angiogenesis, immune evasion, and metastatic spread (Capece et al. 2013; Gonda et al. 2009; Mantovani et al. 2008; Tan et al. 2021). Importantly, this inflammatory tumor microenvironment (TME) also contributes to treatment resistance, making tumors less responsive to conventional therapies (Xia et al. 2020). JAK2/STAT3 is a classical inflammatory pathway that plays an important role in regulating the transition from inflammation to cancer. Rapid and sustained activation of JAK2/STAT3 is closely associated with the onset and progression of HCC and promotes the establishment of the tumor inflammatory microenvironment, which would assist the tumors in attenuating anti‐tumor immune responses, maintaining proliferation, avoiding apoptosis, and accelerating angiogenesis (Zhang et al. 2018). Pro‐inflammatory cytokines, the direct mediators of tumor inflammatory response (Huang et al. 2021), induce functional changes in immune cells and tumor cells, forming a dynamic and complex tumor microenvironment that triggers malignant proliferation, invasion, and metastasis (Fujita et al. 2020; Zeng et al. 2021). Thus, the inflammatory environment, tumor cells, and immune cells are interconnected, and TME, controlling and regulating the survival factors, can directly contribute to tumor formation and progression. Targeting inflammation to treat cancer has been demonstrated in various cancers (Rayburn et al. 2009). Salima et al. (Shebbo et al. 2020) reported that *Matricaria chamomilla* aqueous extract could ameliorate DMH‐induced hepatic damage by inhibiting the Wnt/COX2/iNOS inflammation pathway. In the current study, FPHPE was found to inhibit the growth of HCC by reducing the phosphorylation of JAK2 and STAT3 and preventing STAT3 from going into the nucleus, suggesting that FPHPE alleviated the inflammatory signaling of liver cancer by targeting the JAK2/STAT3 pathway. The in vitro results demonstrated significant cytotoxicity against HCC cell lines, with hallmark apoptotic features confirmed via Annexin V/PI staining and modulation of Bcl‐2 family proteins. In vivo, FPHPE effectively curtailed tumor growth without causing systemic toxicity, underscoring its therapeutic relevance and biocompatibility. Mechanistically, FPHPE inactivated STAT3 signaling by reducing its phosphorylation and nuclear localization, and consequently silenced its downstream targets that govern proliferation (c‐Myc), inflammation (iNOS, COX2), and metastasis (Vimentin, Slug). Apoptosis occurs mainly through receptor and mitochondrial pathways. Bcl2 family proteins, the most important apoptosis regulatory proteins (Ghafouri‐Fard et al. 2021), include the anti‐apoptotic proteins Bcl2, Bcl‐XL, McL‐1, and pro‐apoptotic proteins Bax and Bad, and regulate the mitochondria of apoptotic cells (Radha and Raghavan 2017). Caspase‐3 also plays an irreplaceable role in apoptosis. At the initiation of apoptosis, PARP is cleaved by caspase‐3, resulting in the separation of the two zinc finger structures bound to DNA in PARP from the catalytic region of the carboxyl‐terminal, which prevents PARP from working normally. The cleavage can not only increase Ca^2+^/Mg^2+^‐dependent endonuclease activity, which is negatively regulated by PARP but also cleave the DNA between nucleosomes, leading to apoptosis (De et al. 2018; Kuang et al. 2021). Therefore, promoting tumor cell apoptosis while inhibiting inflammation is regarded as a new strategy for cancer therapy and prevention (Li et al. 2020; Liu et al. 2021). Our study revealed that FPHPE could increase the expressions of apoptotic proteins, including cleaved caspase‐3, cleaved PARP, and Bax, while decreasing the expression of Bcl2, suggesting that FPHPE suppressed HCC growth by inducing apoptosis. In sum, FPHPE markedly suppressed HCC progression through a dual anti‐cancer mechanism substantiated across both cellular and xenograft models. Mechanistically, it reactivated mitochondrial apoptosis via upregulation of cleaved caspase‐3 and PARP, alongside a pro‐apoptotic shift in the Bax/Bcl‐2 ratio. More critically, FPHPE exerted profound anti‐inflammatory effects by disrupting the JAK2/STAT3 signaling cascade—evidenced by reduced phosphorylation of JAK2/STAT3 and inhibited STAT3 nuclear translocation, leading to transcriptional repression of inflammatory and oncogenic effectors including iNOS, COX2, and c‐Myc. These intersecting pathways highlight the extract's ability to simultaneously dismantle pro‐survival and pro‐inflammatory signaling in HCC. As illustrated in Figure 7, molecular docking simulations suggest that key bioactive compounds within FPHPE—such as friedelan‐3‐one and seselin—may bind directly to the ATP‐binding site of JAK2's JH1 domain and the SH2 domain of STAT3, respectively. These interactions are predicted to interfere with phosphorylation and dimerization events essential for STAT3 activation and its downstream inflammatory transcriptional activity. Given its favorable biological profile, FPHPE merits further investigation as a candidate for development into dietary adjuncts or nutraceutical interventions aimed at managing or preventing inflammation‐associated malignancies.

## 5 Conclusions

This study identifies the petroleum ether fraction of *Ficus pandurata* Hance (FPHPE) as a potent dual‐action therapeutic candidate for HCC, acting at the intersection of apoptosis induction and inflammation suppression. Transitioning from a traditional hepatoprotective remedy to a mechanistically validated anti‐cancer agent, FPHPE demonstrated significant efficacy in both in vitro and in vivo models. Importantly, the present work goes beyond conventional assays by incorporating multi‐angle validation of mitochondrial apoptosis, including Annexin V/PI flow cytometry, Western blot quantification from three independent replicates, ultrastructural confirmation of disrupted mitochondrial cristae by TEM, and JC‐1 staining to directly demonstrate mitochondrial membrane depolarization. These convergent findings firmly establish FPHPE as a mitochondria‐targeted apoptotic inducer. In parallel, FPHPE exerted robust inhibition of the JAK2/STAT3 inflammatory signaling cascade, as evidenced by decreased phosphorylation, impaired STAT3 nuclear translocation, and suppression of downstream oncogenic and inflammatory mediators such as c‐Myc, iNOS, COX2, TNF‐α, and IL‐1β. GC–MS profiling revealed a structurally diverse phytochemical composition, with docking simulations suggesting strong binding of key compounds within the JH1 domain of JAK2 and the SH2 domain of STAT3, offering a structural rationale for these biological effects. By simultaneously dismantling pro‐survival and pro‐inflammatory signaling, FPHPE interrupts the pathophysiological loop that sustains HCC progression. Taken together, these findings position FPHPE as a scientifically credible and mechanistically comprehensive phytotherapeutic candidate, with potential applications in the development of functional foods or nutraceutical formulations aimed at combating inflammation‐driven liver cancer. Further standardization, clinical validation, and testing in additional liver cancer models will be the critical next steps toward its translational application.

## Author Contributions

**Muhammad Majid:** data curation (equal), formal analysis (equal), investigation (equal), software (equal), writing – original draft (equal). **Bing Tang:** data curation (equal), formal analysis (equal), investigation (equal), software (equal), visualization (equal), writing – review and editing (equal). **Yingyao Lai:** data curation (equal), software (equal), validation (equal), visualization (equal), writing – review and editing (equal). **Xiaoyan Pang:** formal analysis (equal), investigation (equal), software (equal), visualization (equal), writing – review and editing (equal). **Yongdui Ruan:** formal analysis (equal), methodology (equal), software (equal), writing – review and editing (equal). **Hui Shi:** funding acquisition (equal), methodology (equal), project administration (equal), validation (equal), writing – review and editing (equal). **Weiwen Peng:** funding acquisition (equal), investigation (equal), supervision (equal), validation (equal), writing – review and editing (equal). **Weibo Dai:** conceptualization (equal), funding acquisition (equal), project administration (equal), resources (equal), supervision (equal), writing – review and editing (equal). **Xianjing Hu:** conceptualization (equal), funding acquisition (equal), project administration (equal), resources (equal), writing – review and editing (equal).

## Ethics Statement

The animal experiment was approved by the Animal Ethics and Welfare Committee of Zhongshan Hospital of Traditional Chinese Medicine Affiliated to Guangzhou University of Traditional Chinese Medicine (Approval number: AEWC‐2021057).

## Consent

The authors have nothing to report.

## Conflicts of Interest

The authors declare no conflicts of interest.

## Data Availability

All data are available from the corresponding authors on reasonable request.
